# Hospital-level factors associated with death during pneumonia-associated hospitalization among adults—New York City, 2010–2014

**DOI:** 10.1371/journal.pone.0256678

**Published:** 2021-10-07

**Authors:** Kate Whittemore, Kristian M. Garcia, Chaorui C. Huang, Sungwoo Lim, Demetre C. Daskalakis, Neil M. Vora, David E. Lucero

**Affiliations:** 1 New York City Department of Health and Mental Hygiene, Queens, New York, United States of America; 2 Columbia University Mailman School of Public Health, New York, New York, United States of America; 3 Career Epidemiology Field Officer Program, Centers for Disease Control and Prevention, Atlanta, Georgia, United States of America; University of Notre Dame Australia, AUSTRALIA

## Abstract

**Background:**

In New York City (NYC), pneumonia is a leading cause of death and most pneumonia deaths occur in hospitals. Whether the pneumonia death rate in NYC reflects reporting artifact or is associated with factors during pneumonia-associated hospitalization (PAH) is unknown. We aimed to identify hospital-level factors associated with higher than expected in-hospital pneumonia death rates among adults in NYC.

**Methods:**

Data from January 1, 2010–December 31, 2014 were obtained from the New York Statewide Planning and Research Cooperative System and the American Hospital Association Database. In-hospital pneumonia standardized mortality ratio (SMR) was calculated for each hospital as observed PAH death rate divided by expected PAH death rate. To determine hospital-level factors associated with higher in-hospital pneumonia SMR, we fit a hospital-level multivariable negative binomial regression model.

**Results:**

Of 148,172 PAH among adult NYC residents in 39 hospitals during 2010–2014, 20,820 (14.06%) resulted in in-hospital death. In-hospital pneumonia SMRs varied across NYC hospitals (0.77–1.23) after controlling for patient-level factors. An increase in average daily occupancy and membership in the Council of Teaching Hospitals were associated with increased in-hospital pneumonia SMR.

**Conclusions:**

Differences in in-hospital pneumonia SMRs between hospitals might reflect differences in disease severity, quality of care, or coding practices. More research is needed to understand the association between average daily occupancy and in-hospital pneumonia SMR. Additional pneumonia-specific training at teaching hospitals can be considered to address higher in-hospital pneumonia SMR in teaching hospitals.

## Background

Pneumonia and influenza, often grouped together in vital statistics reports, ranked as the third leading cause of death in NYC in 2017, but the eighth leading cause of death in the United States [1, 2]. Additionally, the age-adjusted death rate for pneumonia and influenza in NYC was nearly double the national death rate in 2016 [1, 2]. In NYC during 1999–2015, pneumonia was listed as the underlying cause of death in 99.3% of all pneumonia and influenza deaths [3]. Furthermore, most pneumonia deaths occur in hospitals; thus, it is important to examine pneumonia-associated hospitalizations (PAH) to better understand patterns of pneumonia mortality. While PAH rates are similar in NYC and the United States, the proportion of in-hospital death during PAH is higher in NYC [4, 5]. It is unclear whether the higher pneumonia death rate in NYC reflects a reporting artifact or is associated with factors during a PAH. One potential contributing factor that has received attention is quality of care [6–9].

A necessary step in any study of quality of care and in-hospital death is controlling for immutable patient-level risk factors that make up a unique hospital case mix, such as age, severity of illness, and pre-existing conditions [10, 11]. Failing to do so in an observational study intended to compare quality of care between hospitals can result in confounding bias. Opportunities for this form of bias can be reduced through the use of accurate risk prediction models that adjust for patient-level factors prior to exploring hospital-level factors (e.g., hospital size, patient volume, number of beds) [12]. Hospital-level characteristics may be associated with patient outcomes and are therefore critical to understanding patterns of in-hospital death from pneumonia [13]. A standardized mortality rate (SMR) model is an example of a prediction model that controls for patient-level factors [14].

Identifying hospital-level risk factors for in-hospital death during PAH can guide public health interventions to address NYC’s reported disproportionate pneumonia burden. The objective of this study was therefore to identify hospital-level factors significantly associated with higher than expected pneumonia death rates in NYC hospitals among adults.

## Methods

### Data sources

Data sources included New York’s Statewide Planning and Research Cooperative System (SPARCS) and the American Hospital Association (AHA) Annual Survey Database [15, 16].

SPARCS is an all-payer reporting system that includes patient-level data on all inpatient and outpatient hospital visits in New York State [15]. SPARCS comprises data on patient demographics, principal and secondary diagnoses, discharge status (e.g., expired), admission dates, discharge dates, and other administrative information pertaining to a hospitalization. Principal and secondary diagnoses were coded using the International Classification of Diseases, Ninth Edition, Clinical Modification (ICD-9-CM) during 2010–2014 [17]. A principal diagnosis code is the condition primarily responsible for the patient’s admission to the hospital for care. Each record also contains fields for up to 24 other secondary diagnoses, which are other conditions present on admission or that developed after admission potentially affecting the treatment received or length of stay.

The AHA Annual Survey Database is compiled from the AHA Annual Survey, which collects data on >6,300 hospitals across the United States [16]. The AHA Annual Survey Database includes hospital-level data on executive contracts and associations, beds and utilization, hospitals and services, insurance, Medicare and Medicaid utilization, and staffing. One survey is completed per group of affiliated hospitals.

### Study population

We identified the discharge records of all NYC residents aged ≥18 years with ≥1 PAH at a NYC hospital who were discharged during January 1, 2010–December 31, 2014. We excluded discharge records with a disposition status indicating that the patient was transferred to a different hospital for inpatient care, as we could not assess outcome for these patients across the continuum of care. If a patient had >1 PAH during the study period, one hospitalization record was randomly selected for inclusion in the analysis [18, 19].

### Definitions

A PAH was defined as a hospitalization with a principal diagnosis of pneumonia (ICD-9-CM codes 480–486, 487.0, 488.01, 488.11, or 488.81) or a secondary diagnosis of pneumonia if the principal diagnosis was respiratory failure (ICD-9-CM codes 518.81, 518.82, 518.84, or 799.1) or sepsis (ICD-9-CM codes 038, 785.52, 995.91, or 995.92) [18]. Pneumonia severity was defined as non-severe if the principal diagnosis was pneumonia or severe if the principal diagnosis was respiratory failure or sepsis and the secondary diagnosis was pneumonia. In-hospital death during PAH was defined as any PAH with a disposition status of “expired”. Observed deaths during PAH were defined as the actual number of patients who died in the hospital during a PAH. Expected deaths during PAH were defined as the predicted number of patients who would die during PAH based on a patient-level prediction model.

Average daily census was defined as the average number of patients of all ages served on an inpatient basis on a single day during the year; the figure was calculated by dividing the number of inpatient days (from the AHA Annual Survey Database) by the number of days in the year [19]. The total number of beds included all beds regularly maintained for inpatients at the close of the annual reporting period and excluded newborn bassinets. We therefore defined average daily occupancy as the ratio of the average daily census to the total number of beds.

Teaching hospitals were defined as hospitals affiliated with a medical school that provide medical education. Major teaching hospitals were defined as members of the Council of Teaching Hospitals (COTH) [19]. COTH is a group of approximately 400 of the nation’s leading teaching hospitals and health systems [20].

### Data analysis

We used R (Vienna, Austria) (packages: tidyverse, MASS, ggplot2, htmlTable, stargazer) for all data analyses and table and figure development [15].

#### Calculation of standardized mortality ratio

We employed a model to determine hospital-level factors associated with in-hospital pneumonia SMR. Before building the model, we calculated the pneumonia SMR for each hospital over the five-year study period. Observed PAH death rates at each hospital were calculated by dividing the actual number of PAH deaths at the hospital during the five-year study period by the number of PAH patients at the hospital during the five-year study period. Expected death rates were calculated by predicting death outcomes for each patient with a PAH in the SPARCS dataset using the patient-level prediction model and then aggregating by hospital. To determine the in-hospital pneumonia SMR by hospital, the observed PAH death rate was divided by the expected PAH death rate. An SMR >1.0 indicates that there were more observed deaths than expected deaths, an SMR <1.0 indicates that there were fewer observed deaths than expected deaths, and an SMR = 1.0 indicates that there were equal numbers of observed deaths and expected deaths.

#### Hospitals

Our SPARCS data containing PAH was reported from 58 hospitals in NYC. After removing 9 nursing homes and end-of-life care hospitals, 49 hospitals remained. We then matched the AHA Annual Survey Database and SPARCS to join hospital-level predictors to our outcome of interest for the hospital-level model, in-hospital pneumonia SMR. Because hospitals in NYC frequently change names and affiliations, we used the Near tool in ArcGIS Pro (Redlands, United States) to facilitate a match between hospitals in the AHA dataset and hospitals in the SPARCS dataset [21]. First, hospitals were mapped to the Geographic Coordinate System World Geodetic System 1984 (EPSG: 4326) using the latitude and longitude provided in the datasets. We then calculated the distance between hospitals in the SPARCS dataset and the nearest hospital in the AHA dataset. Affiliated hospitals from SPARCS were grouped together and joined to their parent hospital in the AHA data. After grouping affiliated hospitals, 39 hospitals were retained in the analysis.

#### Hospital-level model

To identify hospital-level predictors that were independently associated with in-hospital pneumonia SMR, a bivariate analysis was conducted for each candidate predictor of in-hospital pneumonia SMR. Our candidate predictors were chosen based on a literature review and subject matter expertise. For ease of interpretation, our 60 candidate predictors were grouped into categories: executive contracts, beds and utilization, hospitals and services, insurance, Medicare and Medicaid utilization, staffing, and other. Definitions for each of the 60 predictors can be found in the AHA DataViewer Glossary [19].

Candidate predictors with a p-value <0.05 in the bivariate analysis were retained as candidate predictors for the multivariable analysis. We used scatterplots to test whether the relationships between the candidate predictors and the outcome were linear. We used Q–Q plots to test whether the distributions of the variables were normal. To examine multicollinearity between candidate predictors in our multivariable model, we used Chi-square tests, ANOVA tests, and simple linear regression. Several multivariable negative binomial regression models were developed using combinations of variables that were statistically significant (p < 0.05) in the bivariate analysis. We compared these models based on Akaike Information Criterion (AIC) values and chose the model with the lowest AIC value.

#### Study approval

DOHMH determined that this investigation was not human subjects research and CDC determined that this investigation was public health non-research.

## Results

Of 148,172 PAH among adult NYC residents in 39 hospitals during 2010–2014, 20,820 (14.06%) resulted in in-hospital death (Table 1). Of all PAH included in the study, 51.4% were female patients and 48.6% were male patients. Patients aged 65–84 accounted for 40.5% of PAH. More details on PAH by patient characteristic are shown in Table 1.

**Table 1:** Pneumonia-associated hospitalizations (PAH) and in-hospital death by patient characteristics among adults—New York City, 2010–2014*.

	Adult New York City residents at New York City hospitals			
	No. of PAH**	% of PAH	No. of in-hospital deaths	% of PAH with in-hospital death
Sex				
Female	76202	51.43	10621	13.94
Male	71970	48.57	10199	14.17
Age group (years)				
18–44	15028	10.16	589	3.92
45–64	40731	27.54	3705	9.10
65–84	59872	40.48	9327	15.58
≥85	32273	21.82	7191	22.28
Race/Ethnicity				
Hispanic	28027	19.90	3004	10.72
Non-Hispanic American Indian	398	0.28	35	8.79
Non-Hispanic Asian Pacific Islander	8427	5.98	1331	15.79
Non-Hispanic Black	38443	27.30	5053	13.14
Non-Hispanic Other	14517	10.31	1738	11.97
Non-Hispanic White	51007	36.22	8676	17.01
Length of stay (days)				
≤3	30442	22.14	2849	9.36
4–7	47938	34.87	4056	8.46
8–14	33540	24.40	4416	13.17
15–21	12075	8.78	2387	19.77
≥22	13475	9.80	3596	26.69
Primary source of payment				
Medicaid	18928	12.77	1665	8.80
Medicare	87873	59.30	15002	17.07
Other	430	0.29	65	15.12
Private	37492	25.30	3876	10.34
Self-pay	3016	2.04	176	5.84
Unknown	433	0.29	36	8.31
van Walraven comorbidity index score				
≤15	23523	15.88	732	3.11
16–30	84379	56.95	9390	11.13
31–45	37120	25.05	9353	25.20
46–60	3088	2.08	1307	42.33
≥61	62	0.04	38	61.29
Pneumonia severity***				
Non-severe	82038	55.37	3710	4.52
Severe	66134	44.63	17110	25.87
Cancer				
No	127904	86.32	15906	12.44
Yes	20268	13.68	4914	24.25
Heart failure				
No	109272	73.75	13112	12.00
Yes	38900	26.25	7708	19.81
Renal disease				
No	94294	63.64	8139	8.63
Yes	53878	36.36	12681	23.54
Cerebrovascular disease				
No	137958	93.11	18795	13.62
Yes	10214	6.89	2025	19.83
Liver disease				
No	141448	95.46	18792	13.29
Yes	6724	4.54	2028	30.16
Mechanical ventilation				
No	115965	78.26	8360	7.21
Yes	32207	21.74	12460	38.69
Dialysis				
No	138287	93.33	18670	13.50
Yes	9885	6.67	2150	21.75

*Data source: New York’s Statewide Planning and Research Cooperative System (SPARCS).

**Pneumonia-associated hospitalization.

***Pneumonia severity was defined as non-severe if the principal diagnosis was pneumonia or severe if the principal diagnosis was respiratory failure or sepsis and the secondary diagnosis was pneumonia.

In-hospital pneumonia SMR ranged from 0.77–1.23 across 39 hospitals. Of the 39 hospitals, 20 had an SMR >1.0 and 19 had an SMR <1.0. By hospital characteristic, in-hospital pneumonia SMRs ranged from 0.83–1.22 (S2 Table in S1 Appendix). Average daily occupancy ranged from 0.6–1 patient per bed across the 39 hospitals.

Candidate predictors for the hospital-level model are shown in S3 Table in S1 Appendix.

Our final multivariable negative binomial regression model with the lowest AIC score included average daily occupancy and membership in COTH (Table 2). For every 10 unit increase in average daily occupancy (i.e., addition of 10 patients per bed), there was a 26% increase in in-hospital SMR for pneumonia. The ratio of observed to expected deaths increased with an increase in patients per hospital bed. Major teaching hospitals had a 25% higher in-hospital SMR for pneumonia compared to non-teaching hospitals.

**Table 2:** Negative binomial regression model of in-hospital standardized mortality ratio for pneumonia among adults—New York City, 2010–2014.

Hospital-level predictor	Coefficient (95% CI)	p-value
Average daily occupancy * 10	0.230(0.114–0.345)	<0.001
Major teaching hospitals	0.227(0.040–0.416)	0.017
Constant	-1.848(-2.798–-0.887)	<0.001

n = 39; Log Likelihood = -234.363; AIC = 474.726.

## Discussion

Our study objective was to identify hospital-level factors significantly associated with higher than expected pneumonia death rates in NYC hospitals. We found that even after controlling for patient-level factors, hospitals had different in-hospital mortality rates for pneumonia. As average daily occupancy increased, in-hospital mortality rate for pneumonia increased. Major teaching hospitals had higher in-hospital mortality rate for pneumonia when compared with non-teaching hospitals.

We found that an increase in average daily census to bed ratio was associated with an increase in in-hospital pneumonia SMR. Average daily occupancy for NYC hospitals was higher than average daily occupancy for hospitals in the United States, suggesting this factor may play a more important role in NYC. High occupancy has been previously reported in the literature as a risk factor for adverse outcomes in emergency departments [22–24]. One study reported that a 10% increase in emergency department bed relative occupancy ratio was associated with a 3% higher risk of mortality [25]. Another analysis showed that high occupancy in emergency departments was associated with higher 28-day death in community-acquired pneumonia patients [23]. However, our study examined inpatient occupancy. Further research is needed to explore whether improving the average daily occupancy (average daily census to bed ratio) can improve patient outcomes for pneumonia.

Controlling for hospital case mix is particularly important in studies comparing teaching to non-teaching hospitals because sicker patients may be concentrated in teaching hospitals [26]. After controlling for hospital case mix, we found that major teaching hospitals had a higher in-hospital pneumonia SMR compared to non-teaching hospitals. Another recent study showed that death during PAH occurred more frequently during summer months in NYC [4]. This pattern corresponds to the yearly start date of new graduate medical staff (i.e., residents) in the United States. Moreover, New York State had 1507 first year internal medicine residency positions in 2016—the largest number of any state in the country [27]. Further studies can determine whether the large number of graduate medical staff in New York is contributing to higher in-hospital mortality rate for pneumonia in NYC compared with the United States or other factors are involved in the higher mortality rates observed.

Of note, our investigation results differ from a recent study which found that teaching hospitals were associated with lower death rates for common conditions—including pneumonia—compared to non-teaching hospitals [28]. However, that study investigated pneumonia outcomes at hospitals across the United States whereas our study was focused on hospitals in NYC. It should also be noted that this national study examined outcomes among Medicare beneficiaries (aged ≥65 years) while our analysis included outcomes among all adults (aged ≥18 years). Additionally, our definition of pneumonia may have differed from the definition used in the national study.

Our study had several limitations. Our analysis was retrospective and observational, using data not collected specifically for the purpose of this study. There may be biases and changes in coding practices over time for pneumonia in SPARCS [4, 18]. Furthermore, we did not verify the accuracy of SPARCS data. We calculated hospital SMRs over a five-year study period which did not account for potential changes over time during those five years. Additionally, while our study was limited to patients aged ≥18 years, we used a definition of average daily census that included patients of all ages. Data on average daily census stratified by adults and children for each hospital were unavailable. True daily occupancy, which can fluctuate over time, would have been a more robust measure if these data were available. We grouped PAH from affiliated hospitals together to match hospitals in SPARCS with the AHA database. By grouping affiliated hospitals, we likely lost granularity in our outcome. In other words, affiliates of the same hospital group may have had different in-hospital pneumonia SMRs if they had not been grouped together. Finally, our model controlled for the effect of multiple patient-level risk factors; however, there could be additional factors which might have influenced death during PAH.

Despite these limitations, the data presented provide insight into in-hospital death from pneumonia in NYC. While several patient-level models of pneumonia mortality have been previously published, our hospital-level model for pneumonia is the first of its kind, to our knowledge. Differences in in-hospital pneumonia SMRs across hospitals could reflect factors involving patient susceptibility, disease severity, quality of care, or coding practices for which the model did not control. More research is needed to better understand the association between average daily occupancy and in-hospital pneumonia SMR. Major teaching hospitals may benefit from improving pneumonia-specific training for trainees.

## Supporting information

S1 Appendix Supplemental methods and results. (DOCX)

## Abbreviations

AHA: American Hospital Association
COTH: Council of Teaching Hospitals
FT: full-time
FTE: full-time equivalent
ICD-9-CM: *International Classification of Diseases*, *Ninth Revision*, *Clinical Modification*
NYC: New York City
PAH: pneumonia-associated hospitalization
SMR: standardized mortality ratio
SPARCS: Statewide Planning and Research Cooperative System
TE: total equivalent

## References

[1] Centers for Disease Control and Prevention. Mortality in the United States, 2017. NCHS Data Brief https://www.cdc.gov/nchs/products/databriefs/db328.htm

[2] New York City Department of Health and Mental Hygiene. Summary of Vital Statistics 2017. https://www1.nyc.gov/assets/doh/downloads/pdf/vs/2017sum.pdf.

[3] Cordoba E, Maduro G, Huynh M, et al. Deaths from Pneumonia—New York City- 1999–2015. Open Forum Infect Dis. 2018 Jan 16; 5(2). doi: 10.1093/ofid/ofy020 29955618 PMC5824827

[4] Gu CH, Lucero DE, Huang CC, Daskalakis D, Varma JK, Vora NM. Pneumonia-Associated Hospitalizations, New York City, 2001–2014. Publici Health Rep. 2018 Sep/Oct; 133(5): 584–592. doi: 10.1177/0033354918792009 30188808 PMC6134561

[5] Hayes BH, Haberling DL, Kennedy JL, Varma JK, Fry AM, Vora NM. Burden of Pneumonia-Associated Hospitalizations: United States, 2001–2014. Chest. 2018 Feb; 153(2): 427–437. doi: 10.1016/j.chest.2017.09.041 29017956 PMC6556777

[6] Lee JS, Nsa W, Hausmann LR, et al. Quality of care for elderly patients hospitalized for pneumonia in the United States, 2006 to 2010. JAMA Intern Med. 2014 Nov; 174(11): 1806–14. doi: 10.1001/jamainternmed.2014.4501 25201438

[7] Gaskin DJ, Zare H, Haider AH, LaVeist TA. The Quality of Surgical and Pneumonia Care in Minority-Serving and Racially Integrated Hospitals. Health Serv Res. 2016 Jun; 51(3): 910–36. doi: 10.1111/1475-6773.12394 26418717 PMC4874823

[8] Flanagan J, Stamp Kd Fau, Gregas M, Shindul-Rothschild J. Predictors of 30-Day Readmission for Pneumonia. J Nurs Adm. 2016 Feb; 46(2): 69–74. doi: 10.1097/NNA.0000000000000297 26771474

[9] Trivedi AN, Nsa W Fau, Hausmann LRM, Lee JS, et al. Quality and equity of care in U.S. hospitals. N Engl J Med. 2014 Dec 11; 371(24): 2298–308. doi: 10.1056/NEJMsa1405003 25494269

[10] New York State Department of Health. New York State Report on Sepsis Care Improvement Initiative: Hospital Quality Performance. 2017; https://www.health.ny.gov/press/reports/docs/2015_sepsis_care_improvement_initiative.pdf.

[11] Jencks SF, Daley J, Draper D, Thomas N, Lenhart G, Walker J. Interpreting hospital mortality data. The role of clinical risk adjustment. JAMA. 1988 Dec 23–30; 260(24): 3611–6. 3057250

[12] Shine D. Risk-adjusted mortality: problems and possibilities. Comput Math Methods Med. 2012. doi: 10.1155/2012/829465 22474540 PMC3312252

[13] Everitt B, Skrondal, A. The Cambridge dictionary of statistics: Cambridge University Press; 2010:409.

[14] Carretta HJ, Chukmaitov A, Tang A, Shin J. Examination of hospital characteristics and patient quality outcomes using four inpatient quality indicators and 30-day all-cause mortality. Am J Med Qual. 2013 Jan-Feb; 28(1): 46–55. doi: 10.1177/1062860612444459 22723470

[15] Team RC. R: A Language and Environment for Statistical Computing. http://www.R-project.org/.

[16] American Hospital Association Annual Survey Database.

[17] Centers for Disease Control and Prevention. International Classification of Diseases, Ninth Revision, Clinical Modification (ICD-9-CM). https://www.cdc.gov/nchs/icd/icd9cm.htm.

[18] Lindenauer PK, Lagu T, Shieh MS, Pekow PS, Rothberg MB. Association of diagnostic coding with trends in hospitalizations and mortality of patients with pneumonia, 2003–2009. JAMA. 2012 Apr 4; 307(13): 1405–13. doi: 10.1001/jama.2012.384 22474204

[19] American Hospital Association Dataviewer. Glossary https://www.ahadataviewer.com/glossary/.

[20] Council of Teaching Hospitals and Health Systems (COTH). https://www.aamc.org/members/coth/.

[21] Esri. ArcGIS Pro. https://pro.arcgis.com/en/pro-app/.

[22] McCusker J, Vadeboncoeur A, Fau—Levesque J-F, Levesque Jf, Fau—Ciampi A, Ciampi A, Fau—Belzile E, Belzile E. Increases in emergency department occupancy are associated with adverse 30-day outcomes. Acad Emerg Med. 2014 Oct; 21(10): 1092–100. doi: 10.1111/acem.12480 25308131

[23] Jo S, Kim K, Lee JH, Rhee JE, et al. Emergency department crowding is associated with 28-day mortality in community-acquired pneumonia patients. J Infect. 2012 Mar; 64(3): 268–75. doi: 10.1016/j.jinf.2011.12.007 22227383

[24] Jo S, Jin YH, Lee JB, Jeong T, Yoon J, Park B. Emergency department occupancy ratio is associated with increased early mortality. J Emerg Med. 2014 Feb; 46(2): 241–9. doi: 10.1016/j.jemermed.2013.05.026 23992849

[25] van Walraven C, Austin PC, Jennings A, et al. A modification of Elixhauser comorbidity measures into a point system for hospital death using administrative data. Med Care. 2009 Jun; 47(6): 626–33. doi: 10.1097/MLR.0b013e31819432e5 19433995

[26] Ayanian JZ, Weissman JS. Teaching Hospitals and Quality of Care: A Review of the Literature. Milibank Q. 2002; 80(3): 569–93. doi: 10.1111/1468-0009.00023 12233250 PMC2690120

[27] Program NRM. 2016 NRMP Main Residency Match: Match Rates by Specialty and State. 2016; http://www.nrmp.org/wp-content/uploads/2016/04/Main-Match-Results-by-State-and-Specialty-2016.pdf.

[28] Burke LG, Frakt AB, Khullar D, Orav E, Jha AK. Association between teaching status and mortality in us hospitals. *JAMA*. 2017 May; 317(20): 2105–2113. doi: 10.1001/jama.2017.5702 28535236 PMC5815039

