# Supplementary material for: Hospital-level factors associated with death during pneumonia-associated hospitalization among adults—New York City, 2010–2014
Source: PLoS One. 2021 Oct 7;16(10):e0256678. doi: 10.1371/journal.pone.0256678 (PMC8496812; doi:10.1371/journal.pone.0256678)
Supplement: S1 Appendix — (DOCX) [file pone.0256678.s001.docx]

**Appendix**

**Methods**

**Patient-level prediction model**

We used a patient-level prediction model to calculate expected deaths during PAH at a particular NYC hospital. The outcome of interest for our patient-level prediction model was in-hospital death during PAH. Candidate predictors were chosen based on a literature review and subject matter expertise. A van Walraven comorbidity index score was calculated for each patient with a PAH.^23^ We randomly assigned 75% of the patient-level data to a training set and 25% to a testing set. Model performance was assessed using receiver operating characteristic (ROC) curves and c-statistics (equal to the area under the ROC curve); the model with the highest c-statistic was chosen as the final patient-level prediction model. Multicollinearity was assessed with variance inflation factors (VIF). VIFs were calculated from the covariance matrix of parameter estimates using the rms package in R.

**Results**

We included 16 predictors in our final patient-level prediction model (S1 Table). Our final patient-level prediction model had a high level of discrimination (c-statistic = 0.881). Preliminary models which included demographics, comorbidity index scores, and comorbidities/procedures did not perform as well as our final model which included a combination of all these predictors (S1 Fig).

**S1 Table. Final patient-level multivariable model for in-hospital death from pneumonia among adults — New York City, 2010–2014.**

| **Factor** | **OR** | **95% CI** | **p-value** |
| --- | --- | --- | --- |
| Demographics |  |  |  |
| Male | 0.98 | 0.93–1.03 | 0.40 |
| Female (ref.) | ref. | ref. | ref. |
| Age (years) | 1.09 | 1.08–1.09 | <0.001 |
| Non-Hispanic American Indian | 0.97 | 0.58–1.64 | 0.92 |
| Non-Hispanic Asian Pacific Islander | 1.38 | 1.24–1.54 | <0.001 |
| Non-Hispanic Black | 1.11 | 1.03–1.20 | <0.05 |
| Non-Hispanic Other | 0.99 | 0.90–1.08 | 0.77 |
| Non-Hispanic White | 1.34 | 1.25–1.44 | <0.001 |
| Hispanic (ref.) | ref. | ref. | ref. |
| Log length of stay (days) |  |  |  |
| Restricted cubic spline 1 (ref.) | ref. | ref. | ref. |
| Restricted cubic spline 2 | 0.2 | 0.19–0.22 | <0.001 |
| Restricted cubic spline 3 | 3.04 | 2.86–3.23 | 0.001 |
| Comorbidities |  |  |  |
| Comorbidity index score | 1.23 | 1.21–1.25 | <0.001 |
| Severe pneumonia | 57.20 | 40.07–81.64 | <0.001 |
| Cancer | 2.06 | 1.94–2.20 | <0.001 |
| Heart failure | 0.77 | 0.73–0.82 | <0.001 |
| Renal disease | 1.66 | 1.58–1.75 | <0.001 |
| Liver disease | 1.31 | 1.19–1.45 | <0.001 |
| Procedures |  |  |  |
| Mechanical ventilation | 7.23 | 6.83–7.66 | <0.001 |
| Dialysis | 1.28 | 1.18–1.39 | <0.001 |
| Interactions |  |  |  |
| Age x comorbidity index score | 1.00 | 1.00–1.00 | <0.001 |
| Pneumonia severity x comorbidity index score | 0.94 | 0.93–0.94 | <0.001 |
| Age x pneumonia severity | 0.99 | 0.99–0.99 | <0.001 |

ref.: reference group

**S1 Fig. Receiver operating characteristic curves for patient-level prediction model of in-hospital death from pneumonia among adults — New York City, 2010–2014.***


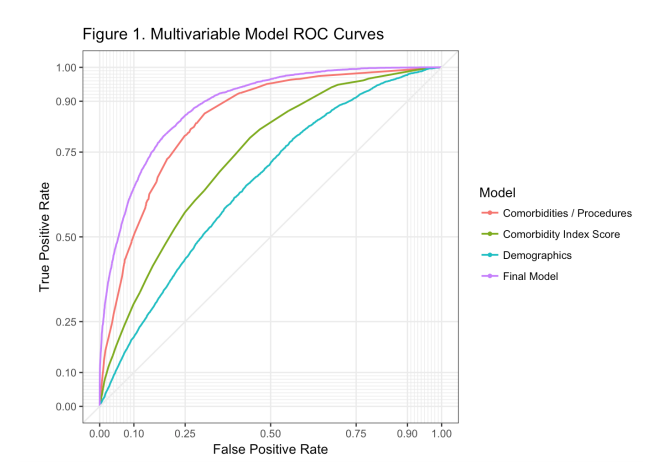


*Data source: New York’s Statewide Planning and Research Cooperative System (SPARCS)

**S2 Table. In-hospital standardized mortality rate for pneumonia by hospital characteristic among adults — New York City, 2010–2014.***

| **Characteristic** | **No. of hospitals** | **No. of PAH**** | **No. of deaths** | **Observed mortality rate** | **Expected mortality rate** | **Standardized mortality rate** |
| --- | --- | --- | --- | --- | --- | --- |
| **Executive contracts and associations** |  |  |  |  |  |  |
| Accreditation Council for Graduate Medical Education residency |  |  |  |  |  |  |
| No | 5 | 12651 | 1909 | 15.09 | 14.39 | 1.05 (1.00–1.10) |
| Yes | 34 | 103051 | 15757 | 15.29 | 14.77 | 1.04 (1.02–1.05) |
| American Medical Association medical school affiliation |  |  |  |  |  |  |
| No | 4 | 7602 | 1201 | 15.8 | 14.14 | 1.12 (1.05–1.18) |
| Yes | 35 | 108100 | 16465 | 15.23 | 14.79 | 1.03 (1.01–1.05) |
| Member of the American Hospital Association |  |  |  |  |  |  |
| No | 9 | 20720 | 3955 | 19.09 | 15.97 | 1.20 (1.16–1.23) |
| Yes | 30 | 94982 | 13711 | 14.44 | 14.35 | 1.01 (0.99–1.02) |
| American Osteopathic Association internship |  |  |  |  |  |  |
| No | 34 | 103275 | 15457 | 14.97 | 14.38 | 1.04 (1.02–1.06) |
| Yes | 5 | 12427 | 2209 | 17.78 | 17.03 | 1.04 (1.00–1.09) |
| Catholic |  |  |  |  |  |  |
| No | 38 | 113456 | 17201 | 15.16 | 14.61 | 1.04 (1.02–1.05) |
| Yes | 1 | 2246 | 465 | 20.7 | 18.93 | 1.09 (1.00–1.20) |
| Member of the Council of Teaching Hospitals of the AAMC |  |  |  |  |  |  |
| No | 25 | 52521 | 8252 | 15.71 | 14.31 | 1.10 (1.07–1.12) |
| Yes | 14 | 63181 | 9414 | 14.9 | 15.45 | 0.96 (0.95–0.98) |
| Joint Commission Accreditation |  |  |  |  |  |  |
| No | 1 | 2113 | 332 | 15.71 | 15.86 | 0.99 (0.89–1.10) |
| Yes | 38 | 113589 | 17334 | 15.26 | 14.69 | 1.04 (1.02–1.05) |
| Teaching affiliation |  |  |  |  |  |  |
| Non-teaching | 4 | 7602 | 1201 | 15.8 | 14.14 | 1.12 (1.05–1.18) |
| Major | 14 | 63181 | 9414 | 14.9 | 15.45 | 0.96 (0.95–0.98) |
| Minor | 21 | 44919 | 7051 | 15.7 | 14.35 | 1.09 (1.07–1.12) |
| **Beds and utilization** |  |  |  |  |  |  |
| Admission to bed ratio |  |  |  |  |  |  |
| 0.125–8.29 | 10 | 23190 | 3543 | 15.28 | 14.45 | 1.06 (1.02–1.09) |
| 8.30–11.0 | 10 | 36581 | 5485 | 14.99 | 15.73 | 0.95 (0.93–0.98) |
| 11.1–13.9 | 9 | 28945 | 4334 | 14.97 | 14.84 | 1.01 (0.98–1.04) |
| 14.0–91.2 | 10 | 26986 | 4304 | 15.95 | 13.88 | 1.15 (1.11–1.18) |
| Average daily occupancy * 10 |  |  |  |  |  |  |
| 6.18–8.14 | 10 | 26601 | 3476 | 13.07 | 13.4 | 0.98 (0.94–1.01) |
| 8.15–8.50 | 10 | 21505 | 3354 | 15.6 | 14.32 | 1.09 (1.05–1.12) |
| 8.51–8.74 | 9 | 23419 | 3882 | 16.58 | 16.65 | 1.00 (0.96–1.03) |
| 8.75–10.00 | 10 | 44177 | 6954 | 15.74 | 14.71 | 1.07 (1.05–1.10) |
| General medical/surgical adult beds |  |  |  |  |  |  |
| 79–170 | 9 | 14040 | 2420 | 17.24 | 14.39 | 1.20 (1.15–1.25) |
| 171–225 | 8 | 20326 | 3113 | 15.32 | 15.48 | 0.99 (0.95–1.02) |
| 226–377 | 8 | 25959 | 3553 | 13.69 | 13.13 | 1.04 (1.01–1.08) |
| 378–1080 | 8 | 41615 | 6189 | 14.87 | 16.45 | 0.90 (0.88–0.93) |
| Hospital size |  |  |  |  |  |  |
| Large | 22 | 86049 | 12855 | 14.94 | 15.1 | 0.99 (0.97–1.01) |
| Medium | 17 | 29653 | 4811 | 16.22 | 14.23 | 1.14 (1.11–1.17) |
| Medical/surgical intensive care beds |  |  |  |  |  |  |
| 7–18 | 10 | 19629 | 3215 | 16.38 | 15.31 | 1.07 (1.03–1.11) |
| 19–22 | 7 | 15202 | 2301 | 15.14 | 14.13 | 1.07 (1.03–1.12) |
| 23–32 | 8 | 23295 | 3373 | 14.48 | 14.98 | 0.97 (0.93–1.00) |
| 33–137 | 8 | 43814 | 6386 | 14.58 | 14.76 | 0.99 (0.96–1.01) |
| Total hospital beds |  |  |  |  |  |  |
| 128–286 | 10 | 16116 | 2464 | 15.29 | 13.24 | 1.15 (1.11–1.20) |
| 287–508 | 10 | 18993 | 3072 | 16.17 | 15.66 | 1.03 (1.00–1.07) |
| 509–730 | 9 | 35034 | 5559 | 15.87 | 15.15 | 1.05 (1.02–1.08) |
| 731–2260 | 10 | 45559 | 6571 | 14.42 | 14.87 | 0.97 (0.95–0.99) |
| **Facilities and services** |  |  |  |  |  |  |
| Airborne infection isolation room |  |  |  |  |  |  |
| No | 2 | 3465 | 404 | 11.66 | 12.59 | 0.93 (0.84–1.02) |
| Yes | 31 | 98475 | 14871 | 15.1 | 14.99 | 1.01 (0.99–1.02) |
| Case management advanced practice nurses |  |  |  |  |  |  |
| No | 21 | 69663 | 10228 | 14.68 | 14.61 | 1.01 (0.99–1.02) |
| Yes | 7 | 24732 | 3693 | 14.93 | 16.13 | 0.93 (0.90–0.96) |
| Emergency department |  |  |  |  |  |  |
| No | 1 | 1210 | 187 | 15.45 | 16.82 | 0.92 (0.79–1.06) |
| Yes | 32 | 100730 | 15088 | 14.98 | 14.79 | 1.01 (1.00–1.03) |
| Emergency department care advanced practice nurses |  |  |  |  |  |  |
| No | 10 | 24561 | 3950 | 16.08 | 15.12 | 1.06 (1.03–1.10) |
| Yes | 18 | 69834 | 9971 | 14.28 | 14.91 | 0.96 (0.94–0.98) |
| HIV/AIDS services |  |  |  |  |  |  |
| No | 3 | 6368 | 1020 | 16.02 | 14.71 | 1.09 (1.02–1.16) |
| Yes | 30 | 95572 | 14255 | 14.92 | 14.86 | 1.00 (0.99–1.02) |
| Hospitalists provide care |  |  |  |  |  |  |
| No | 1 | 2049 | 359 | 17.52 | 17.77 | 0.99 (0.89–1.09) |
| Yes | 30 | 96724 | 14321 | 14.81 | 14.7 | 1.01 (0.99–1.02) |
| Intensivists provide care |  |  |  |  |  |  |
| No | 2 | 4487 | 1056 | 23.53 | 19.65 | 1.20 (1.13–1.27) |
| Yes | 27 | 89038 | 12888 | 14.47 | 14.56 | 0.99 (0.98–1.01) |
| Number of airborne infection isolation rooms |  |  |  |  |  |  |
| 3–22 | 8 | 30259 | 4725 | 15.62 | 16.38 | 0.95 (0.93–0.98) |
| 23–51 | 7 | 18520 | 2782 | 15.02 | 14.23 | 1.06 (1.02–1.10) |
| 52–87 | 8 | 22771 | 3661 | 16.08 | 15.92 | 1.01 (0.98–1.04) |
| 88–120 | 6 | 22380 | 3140 | 14.03 | 13.77 | 1.02 (0.98–1.06) |
| Other specialty care advanced practice nurses |  |  |  |  |  |  |
| No | 7 | 21507 | 3265 | 15.18 | 15.38 | 0.99 (0.95–1.02) |
| Yes | 21 | 72888 | 10656 | 14.62 | 14.86 | 0.98 (0.97–1.00) |
| Patient education advanced practice nurses |  |  |  |  |  |  |
| No | 23 | 80024 | 11764 | 14.7 | 14.72 | 1.00 (0.98–1.02) |
| Yes | 5 | 14371 | 2157 | 15.01 | 16.23 | 0.92 (0.89–0.96) |
| Primary care advanced practice nurses |  |  |  |  |  |  |
| No | 4 | 11505 | 1874 | 16.29 | 15.24 | 1.07 (1.02–1.12) |
| Yes | 24 | 82890 | 12047 | 14.53 | 14.94 | 0.97 (0.96–0.99) |
| Primary care department |  |  |  |  |  |  |
| No | 3 | 4377 | 782 | 17.87 | 15.98 | 1.12 (1.04–1.20) |
| Yes | 30 | 97563 | 14493 | 14.86 | 14.74 | 1.01 (0.99–1.02) |
| Total gross square feet by daily census |  |  |  |  |  |  |
| 735–1800 | 8 | 22231 | 3746 | 16.85 | 17.43 | 0.97 (0.94–1.00) |
| 1810–2390 | 8 | 22386 | 3886 | 17.36 | 15.98 | 1.09 (1.05–1.12) |
| 2400–3510 | 7 | 28375 | 3634 | 12.81 | 12.42 | 1.03 (1.00–1.07) |
| 3520–5040 | 8 | 24413 | 3297 | 13.51 | 13.79 | 0.98 (0.95–1.01) |
| **Insurance** |  |  |  |  |  |  |
| Blue Cross participant |  |  |  |  |  |  |
| No | 1 | 2113 | 332 | 15.71 | 15.86 | 0.99 (0.87–1.10) |
| Yes | 38 | 113589 | 17334 | 15.26 | 14.69 | 1.04 (1.02–1.05) |
| Health maintenance organization |  |  |  |  |  |  |
| No | 14 | 44815 | 6946 | 15.50 | 14.98 | 1.03 (1.01–1.06) |
| Yes | 19 | 57125 | 8329 | 14.58 | 14.75 | 0.99 (0.97–1.01) |
| Indemnity fee for service plan |  |  |  |  |  |  |
| No | 30 | 97511 | 14459 | 14.83 | 14.83 | 1.00 (0.98–1.02) |
| Yes | 3 | 4429 | 816 | 18.42 | 15.06 | 1.22 (1.14–1.31) |
| Paid on capitated basis |  |  |  |  |  |  |
| 0–1.50 | 15 | 51093 | 7778 | 15.22 | 15.46 | 0.98 (0.96–1.01) |
| 1.51–3.75 | 7 | 19036 | 2795 | 14.68 | 14.13 | 1.04 (1.00–1.08) |
| 3.76–31.00 | 8 | 27613 | 4322 | 15.65 | 16.08 | 0.97 (0.94–1.00) |
| Paid on shared risk basis |  |  |  |  |  |  |
| 0–0.50 | 15 | 54381 | 8525 | 15.68 | 16.22 | 0.97 (0.95–0.99) |
| 0.51–3.75 | 7 | 25646 | 3432 | 13.38 | 13.41 | 1.00 (0.96–1.03) |
| 3.76–23 | 8 | 18025 | 2830 | 15.7 | 14.51 | 1.08 (1.04–1.12) |
| Preferred provider organization |  |  |  |  |  |  |
| No | 30 | 98362 | 14790 | 15.04 | 15.07 | 1.00 (0.98–1.01) |
| Yes | 3 | 3578 | 485 | 13.56 | 12.62 | 1.07 (0.98–1.17) |
| **Medicare and Medicaid utilization** |  |  |  |  |  |  |
| Total Medicaid days |  |  |  |  |  |  |
| 7920–21000 | 10 | 16775 | 2538 | 15.13 | 13.8 | 1.10 (1.05–1.14) |
| 21100–55900 | 10 | 29989 | 5013 | 16.72 | 15.26 | 1.10 (1.07–1.13) |
| 56000–99800 | 9 | 26019 | 3953 | 15.19 | 15.28 | 0.99 (0.96–1.03) |
| 99900–222000 | 10 | 42919 | 6162 | 14.36 | 14.6 | 0.98 (0.96–1.01) |
| Total Medicaid discharge to admission ratio |  |  |  |  |  |  |
| 0.0883–1.06 | 10 | 24092 | 3730 | 15.48 | 13.99 | 1.11 (1.07–1.14) |
| 1.07–2.10 | 10 | 36926 | 5851 | 15.85 | 16.18 | 0.98 (0.95–1.00) |
| 2.11–2.55 | 9 | 20111 | 3229 | 16.06 | 14.76 | 1.09 (1.05–1.12) |
| 2.56–114 | 10 | 34573 | 4856 | 14.05 | 13.95 | 1.01 (0.98–1.04) |
| Total Medicare days |  |  |  |  |  |  |
| 19100–36500 | 10 | 14478 | 1954 | 13.5 | 12.71 | 1.06 (1.02–1.11) |
| 36600–44700 | 10 | 22494 | 3563 | 15.84 | 14.83 | 1.07 (1.03–1.10) |
| 44800–84500 | 9 | 24483 | 4113 | 16.8 | 15.91 | 1.06 (1.02–1.09) |
| 84600–271000 | 10 | 54247 | 8036 | 14.81 | 15.55 | 0.95 (0.93–0.97) |
| Total Medicare discharge to admission ratio |  |  |  |  |  |  |
| 0.238–0.915 | 10 | 20213 | 2686 | 13.29 | 12.3 | 1.08 (1.04–1.12) |
| 0.916–1.520 | 10 | 18450 | 2842 | 15.4 | 15.48 | 1.00 (0.96–1.03) |
| 1.521–1.780 | 9 | 33365 | 5643 | 16.91 | 16.55 | 1.02 (1.00–1.05) |
| 1.781–137.000 | 10 | 43674 | 6495 | 14.87 | 14.74 | 1.01 (0.98–1.03) |
| **Staffing** |  |  |  |  |  |  |
| Advanced practice nurse (FT^†^) to bed ratio |  |  |  |  |  |  |
| 0.00455–0.0269 | 8 | 18647 | 2671 | 14.32 | 14.48 | 0.99 (0.95–1.03) |
| 0.0270–0.0771 | 7 | 22857 | 3731 | 16.32 | 14.65 | 1.11 (1.08–1.15) |
| 0.0772–0.1330 | 7 | 21113 | 3017 | 14.29 | 15 | 0.95 (0.92–0.99) |
| 0.1331–0.783 | 7 | 32520 | 4536 | 13.95 | 14.78 | 0.94 (0.92–0.97) |
| Emergency medicine physician (TE^‡^) to bed ratio |  |  |  |  |  |  |
| 0–0.00227 | 7 | 12547 | 1979 | 15.77 | 14.79 | 1.07 (1.02–1.11) |
| 0.00228–0.06220 | 7 | 15079 | 2089 | 13.85 | 13.83 | 1.00 (0.96–1.05) |
| 0.06221–0.08940 | 7 | 32869 | 5028 | 15.3 | 15.98 | 0.96 (0.93–0.98) |
| 0.08941–0.126 | 7 | 24202 | 3634 | 15.02 | 15.21 | 0.99 (0.95–1.02) |
| Hospitalist (FTE^§^) to bed ratio |  |  |  |  |  |  |
| 0.0013–0.0438 | 8 | 36328 | 4995 | 13.75 | 14.79 | 0.93 (0.90–0.96) |
| 0.0439–0.0759 | 8 | 27074 | 3865 | 14.28 | 14.56 | 0.98 (0.95–1.01) |
| 0.0760–0.1780 | 7 | 18413 | 2973 | 16.15 | 15.23 | 1.06 (1.02–1.10) |
| 0.1781–0.344 | 8 | 16958 | 2847 | 16.79 | 14.68 | 1.14 (1.10–1.19) |
| Intensivist (TE^‡^) to bed ratio |  |  |  |  |  |  |
| 0–0.0247 | 14 | 34259 | 5407 | 15.78 | 14.98 | 1.05 (1.03–1.08) |
| 0.0248–0.0315 | 7 | 28403 | 4283 | 15.08 | 16.33 | 0.92 (0.90–0.95) |
| 0.0316–0.0736 | 7 | 22035 | 3040 | 13.8 | 13.52 | 1.02 (0.98–1.06) |
| Laboratory technician (FTE^§^) to bed ratio |  |  |  |  |  |  |
| 0–0.0712 | 8 | 16649 | 2598 | 15.6 | 14.32 | 1.09 (1.05–1.13) |
| 0.0713–0.104 | 7 | 21740 | 3463 | 15.93 | 16.39 | 0.97 (0.94–1.00) |
| 0.105–0.178 | 7 | 23502 | 3571 | 15.19 | 14.83 | 1.02 (0.99–1.06) |
| 0.179–0.548 | 8 | 36569 | 5127 | 14.02 | 15.23 | 0.92 (0.90–0.95) |
| Medical surgical intensive care physician (FTE^§^) to bed ratio |  |  |  |  |  |  |
| 0–0.0081 | 8 | 21387 | 3167 | 14.81 | 15.04 | 0.98 (0.95–1.02) |
| 0.0082–0.0128 | 7 | 17872 | 2532 | 14.17 | 14.46 | 0.98 (0.94–1.02) |
| 0.0129–0.0172 | 7 | 22640 | 3614 | 15.96 | 15.93 | 1.00 (0.97–1.04) |
| 0.0173–0.0338 | 8 | 34372 | 4829 | 14.05 | 13.39 | 1.05 (1.02–1.08) |
| Pharmacist (FT^†^) to bed ratio |  |  |  |  |  |  |
| 0.0227–0.0446 | 10 | 30326 | 5483 | 18.08 | 17.45 | 1.04 (1.01–1.06) |
| 0.0447–0.0625 | 10 | 36630 | 5205 | 14.21 | 13.96 | 1.02 (0.99–1.05) |
| 0.0626–0.090 | 9 | 20479 | 3249 | 15.87 | 13.78 | 1.15 (1.11–1.19) |
| 0.091–0.448 | 10 | 28267 | 3729 | 13.19 | 13.6 | 0.97 (0.94–1.00) |
| Pharmacy technician (FT^†^) to bed ratio |  |  |  |  |  |  |
| 0–0.0291 | 10 | 23611 | 3834 | 16.24 | 14.96 | 1.09 (1.05–1.12) |
| 0.0292–0.0381 | 10 | 24960 | 4141 | 16.59 | 15.45 | 1.07 (1.04–1.11) |
| 0.0382–0.0546 | 9 | 35217 | 5088 | 14.45 | 14.55 | 0.99 (0.97–1.02) |
| 0.0547–0.186 | 10 | 31914 | 4603 | 14.42 | 13.9 | 1.04 (1.01–1.07) |
| Physicians and dentist (FT^†^) to bed ratio |  |  |  |  |  |  |
| 0–0.065 | 10 | 28897 | 3922 | 13.57 | 13.79 | 0.98 (0.95–1.02) |
| 0.066–0.126 | 10 | 21469 | 3510 | 16.35 | 15.16 | 1.08 (1.04–1.11) |
| 0.127–0.333 | 9 | 27312 | 4796 | 17.56 | 15.58 | 1.13 (1.10–1.16) |
| 0.334–1.41 | 10 | 38024 | 5438 | 14.3 | 14.44 | 0.99 (0.96–1.02) |
| Primary care physician (TE^‡^) to bed ratio |  |  |  |  |  |  |
| 0–0.0303 | 7 | 13152 | 2259 | 17.18 | 16.32 | 1.05 (1.01–1.10) |
| 0.0304–0.1360 | 7 | 17644 | 2356 | 13.35 | 12.83 | 1.04 (1.00–1.08) |
| 0.1361–0.2110 | 7 | 25439 | 4080 | 16.04 | 16.84 | 0.95 (0.92–0.98) |
| 0.2111–0.4490 | 7 | 28462 | 4035 | 14.18 | 13.83 | 1.03 (0.99–1.06) |
| Radiologist, pathologist, and anesthesiologist (TE^‡^) to bed ratio |  |  |  |  |  |  |
| 0–0.00218 | 7 | 18110 | 2573 | 14.21 | 14.45 | 0.98 (0.95–1.02) |
| 0.00219–0.05130 | 7 | 20259 | 2988 | 14.75 | 14.02 | 1.05 (1.01–1.09) |
| 0.05131–0.10400 | 7 | 18769 | 3036 | 16.18 | 15.23 | 1.06 (1.02–1.10) |
| 0.10401–0.36700 | 7 | 27559 | 4133 | 15 | 16.1 | 0.93 (0.90–0.96) |
| Radiology technician (FT^†^) to bed ratio |  |  |  |  |  |  |
| 0–0.0571 | 10 | 22469 | 3562 | 15.85 | 14.15 | 1.12 (1.08–1.16) |
| 0.0572–0.1240 | 10 | 22977 | 3663 | 15.94 | 15.83 | 1.01 (0.97–1.04) |
| 0.1241–0.1710 | 9 | 31927 | 5134 | 16.08 | 15.1 | 1.06 (1.04–1.09) |
| 0.1711–0.7680 | 10 | 38329 | 5307 | 13.85 | 13.85 | 1.00 (0.97–1.03) |
| Registered nurse (FT^†^) to bed ratio |  |  |  |  |  |  |
| 0.388–1.230 | 10 | 20538 | 3405 | 16.58 | 15.43 | 1.07 (1.04–1.11) |
| 1.231–1.630 | 10 | 28580 | 4748 | 16.61 | 13.99 | 1.19 (1.15–1.22) |
| 1.631–1.870 | 9 | 36638 | 5484 | 14.97 | 15.48 | 0.97 (0.94–0.99) |
| 1.871–3.940 | 10 | 29946 | 4029 | 13.45 | 14.06 | 0.96 (0.93–0.99) |
| Respiratory therapist (FT^†^) to bed ratio |  |  |  |  |  |  |
| 0.0185–0.0471 | 10 | 24793 | 3708 | 14.96 | 14.42 | 1.04 (1.00–1.07) |
| 0.0472–0.0638 | 10 | 33204 | 4804 | 14.47 | 14.29 | 1.01 (0.98–1.04) |
| 0.0639–0.0805 | 9 | 37020 | 5849 | 15.8 | 16.24 | 0.97 (0.95–1.00) |
| 0.0806–0.155 | 10 | 20685 | 3305 | 15.98 | 14.09 | 1.13 (1.10–1.17) |
| Total physician (TE^‡^) to bed ratio |  |  |  |  |  |  |
| 0–0.144 | 7 | 13386 | 2151 | 16.07 | 14.93 | 1.08 (1.03–1.12) |
| 0.145–0.599 | 7 | 21360 | 3264 | 15.28 | 14.36 | 1.06 (1.03–1.10) |
| 0.600–0.843 | 7 | 17393 | 2753 | 15.83 | 15.85 | 1.00 (0.96–1.04) |
| 0.844–1.430 | 7 | 32558 | 4562 | 14.01 | 14.67 | 0.95 (0.93–0.98) |
| **Other** |  |  |  |  |  |  |
| Diversity orientation for clinical staff |  |  |  |  |  |  |
| No | 1 | 2502 | 538 | 21.5 | 18.75 | 1.15 (1.05–1.25) |
| Yes | 31 | 98517 | 14607 | 14.83 | 14.81 | 1.00 (0.98–1.02) |
| Diversity strategy plan |  |  |  |  |  |  |
| No | 2 | 5435 | 968 | 17.81 | 18.16 | 0.98 (0.92–1.04) |
| Yes | 30 | 95584 | 14177 | 14.83 | 14.72 | 1.01 (0.99–1.02) |
| Diversity training for all employees is required |  |  |  |  |  |  |
| No | 1 | 2933 | 430 | 14.66 | 17.58 | 0.83 (0.76–0.92) |
| Yes | 31 | 98086 | 14715 | 15 | 14.85 | 1.01 (0.99–1.03) |
| Hospital has electronic health record |  |  |  |  |  |  |
| Yes, fully implemented | 21 | 69204 | 9657 | 13.95 | 13.85 | 1.01 (0.99–1.03) |
| Yes, partially implemented | 11 | 31815 | 5488 | 17.25 | 17 | 1.01 (0.99–1.04) |
| More foreign educated nurses hired to fill registered nurse vacancies |  |  |  |  |  |  |
| Did not hire foreign nurses | 24 | 71591 | 10721 | 14.98 | 15.06 | 0.99 (0.95–1.03) |
| Less | 1 | 5031 | 916 | 18.21 | 16.46 | 1.11 (1.04–1.18) |
| More | 1 | 2711 | 280 | 10.33 | 11.44 | 0.90 (0.80–1.02) |
| Same | 3 | 15439 | 2401 | 15.55 | 15.69 | 0.99 (0.95–1.03) |
| Number of new registered nurse graduates hired by bed |  |  |  |  |  |  |
| 0–0.0145 | 8 | 18545 | 2983 | 16.09 | 15.05 | 1.07 (1.03–1.11) |
| 0.0146–0.0425 | 7 | 16056 | 2321 | 14.46 | 14.68 | 0.98 (0.95–1.03) |
| 0.0426–0.0858 | 7 | 26180 | 3543 | 13.53 | 13.57 | 1.00 (0.96–1.03) |
| 0.0859–0.213 | 8 | 35490 | 5529 | 15.58 | 16.65 | 0.94 (0.91–0.96) |
| Strategic plan includes quality improvement goals for diverse patient populations |  |  |  |  |  |  |
| No | 1 | 3614 | 582 | 16.1 | 13.27 | 1.21 (1.12–1.32) |
| Yes | 31 | 97405 | 14563 | 14.95 | 14.99 | 1.00 (0.98–1.01) |

*Data sources: New York’s Statewide Planning and Research Cooperative System (SPARCS) and American Hospital Association (AHA) Annual Survey Database

**Pneumonia-associated hospitalization

^†^Full-time

^‡^Total equivalent

^§^Full-time equivalent

**S3 Table. Bivariate analysis of predictors of in-hospital standardized mortality rate for pneumonia among adults — New York City, 2010–2014.***

| **Characteristic** | **Estimate** | **Standard error** | **z-value** | **p-value** |
| --- | --- | --- | --- | --- |
| **Executive contracts and associations** |  |  |  |  |
| Accreditation Council for Graduate Medical Education residency |  |  |  |  |
| Yes | 0.06 | 0.17 | 0.34 | 0.733 |
| AMA medical school affiliation |  |  |  |  |
| Yes | 0.11 | 0.19 | 0.57 | 0.565 |
| Member of the American Hospital Association |  |  |  |  |
| Yes | 0.03 | 0.14 | 0.19 | 0.846 |
| American Osteopathic Association internship |  |  |  |  |
| Yes | -0.19 | 0.17 | -1.14 | 0.255 |
| American Osteopathic Association residency |  |  |  |  |
| Yes | -0.26 | 0.14 | -1.81 | 0.071 |
| Catholic |  |  |  |  |
| Yes | -0.1 | 0.36 | -0.27 | 0.785 |
| Member of the Council of Teaching Hospitals |  |  |  |  |
| Yes | 0.33 | 0.11 | 3.07 | <0.001 |
| Joint Commission Accreditation |  |  |  |  |
| Yes | 0.2 | 0.36 | 0.55 | 0.581 |
| Teaching affiliation |  |  |  |  |
| Major | 0.3 | 0.18 | 1.62 | 0.106 |
| Minor | -0.04 | 0.18 | -0.23 | 0.821 |
| **Beds and utilization** |  |  |  |  |
| Admission to bed ratio |  |  |  |  |
| Admission to bed ratio | 0 | 0 | -0.84 | 0.402 |
| Average daily occupancy |  |  |  |  |
| Average daily occupancy | 0.27 | 0.06 | 4.26 | <0.001 |
| General medical/surgical adult beds |  |  |  |  |
| General medical/surgical adult beds | 0 | 0 | 3.5 | <0.001 |
| Hospital size |  |  |  |  |
| Medium | -0.21 | 0.11 | -1.89 | 0.058 |
| Medical/surgical intensive care beds |  |  |  |  |
| Medical/surgical intensive care beds | 0.01 | 0 | 2.7 | 0.007 |
| Total hospital beds |  |  |  |  |
| Total hospital beds | 0 | 0 | 3.24 | <0.001 |
| **Facilities and services** |  |  |  |  |
| Airborne infection isolation room |  |  |  |  |
| Yes | 0.21 | 0.28 | 0.75 | 0.454 |
| Case management advanced practice nurses |  |  |  |  |
| Yes | -0.13 | 0.18 | -0.73 | 0.464 |
| Emergency department |  |  |  |  |
| Yes | 0.28 | 0.4 | 0.72 | 0.473 |
| Emergency department care advanced practice nurses |  |  |  |  |
| Yes | 0.3 | 0.15 | 1.95 | 0.051 |
| HIV/AIDS services |  |  |  |  |
| Yes | 0.14 | 0.24 | 0.61 | 0.541 |
| Hospitalists provide care |  |  |  |  |
| Yes | 0.22 | 0.41 | 0.53 | 0.593 |
| Intensivists provide care |  |  |  |  |
| Yes | -0.05 | 0.27 | -0.18 | 0.861 |
| Number of airborne infection isolation rooms |  |  |  |  |
| Number of airborne infection isolation rooms | 0 | 0 | -0.38 | 0.702 |
| Other specialty care advanced practice nurses |  |  |  |  |
| Yes | -0.02 | 0.18 | -0.12 | 0.901 |
| Patient education advanced practice nurses |  |  |  |  |
| Yes | -0.38 | 0.19 | -1.97 | 0.048 |
| Primary care advanced practice nurses |  |  |  |  |
| Yes | 0.16 | 0.22 | 0.74 | 0.459 |
| Primary care department |  |  |  |  |
| Yes | 0.15 | 0.24 | 0.63 | 0.526 |
| Total gross square feet by daily census |  |  |  |  |
| Total gross square feet by daily census | 0 | 0 | 1.4 | 0.161 |
| **Insurance** |  |  |  |  |
| Blue Cross participant |  |  |  |  |
| Yes | 0.2 | 0.36 | 0.55 | 0.581 |
| Health maintenance organization |  |  |  |  |
| Yes | -0.05 | 0.14 | -0.36 | 0.716 |
| Indemnity fee for service plan |  |  |  |  |
| Yes | -0.27 | 0.24 | -1.16 | 0.247 |
| Paid on capitated basis |  |  |  |  |
| Paid on capitated basis | 0 | 0.01 | 0.54 | 0.587 |
| Paid on shared risk basis |  |  |  |  |
| Paid on shared risk basis | -0.01 | 0.01 | -0.92 | 0.357 |
| Preferred provider organization |  |  |  |  |
| Yes | -0.29 | 0.24 | -1.21 | 0.227 |
| **Medicare and Medicaid utilization** |  |  |  |  |
| Total Medicaid days |  |  |  |  |
| Total Medicaid days | 0 | 0 | 2.36 | 0.018 |
| Total Medicaid discharge to admission ratio |  |  |  |  |
| Total Medicaid discharges by admissions | 0.01 | 0 | 1.77 | 0.077 |
| Total Medicare days |  |  |  |  |
| Total Medicare days | 0 | 0 | 4.71 | <0.001 |
| Total Medicare discharge to admission ratio |  |  |  |  |
| Total Medicare discharge to admission ratio | 0 | 0 | 1.84 | 0.065 |
| **Staffing** |  |  |  |  |
| Advanced practice nurse (FT^†^) to bed ratio |  |  |  |  |
| Advanced practice nurse (FT^†^) to bed ratio | 0.34 | 0.45 | 0.74 | 0.458 |
| Emergency medicine physician (TE^‡^) to bed ratio |  |  |  |  |
| Emergency medicine physician (TE^‡^) to bed ratio | 5.19 | 1.48 | 3.51 | <0.001 |
| Hospitalist (FTE^§^) to bed ratio |  |  |  |  |
| Hospitalist (FTE^§^) to bed ratio | -1.2 | 0.75 | -1.61 | 0.108 |
| Intensivist (TE^‡^) to bed ratio |  |  |  |  |
| Intensivist (TE^‡^) to bed ratio | 9.34 | 3.71 | 2.52 | 0.012 |
| Laboratory technician (FTE^§^) to bed ratio |  |  |  |  |
| Laboratory technician (FTE^§^) to bed ratio | 0.85 | 0.61 | 1.4 | 0.162 |
| Medical surgical intensive care physician (FTE^§^) to bed ratio |  |  |  |  |
| Medical surgical intensive care physician (FTE^§^) to bed ratio | 20.5 | 10.17 | 2.02 | 0.044 |
| Pharmacist (FT^†^) to bed ratio |  |  |  |  |
| Pharmacist (FT^†^) to bed ratio | -0.78 | 0.83 | -0.95 | 0.343 |
| Pharmacy technician (FT^†^) to bed ratio |  |  |  |  |
| Pharmacy technician (FT^†^) to bed ratio | 2.6 | 1.81 | 1.43 | 0.151 |
| Physician and dentist (FT^†^) to bed ratio |  |  |  |  |
| Physician and dentist (FT^†^) to bed ratio | 0.33 | 0.16 | 1.99 | 0.046 |
| Primary care physician (TE^‡^) to bed ratio |  |  |  |  |
| Primary care physician (TE^‡^) to bed ratio | 1.63 | 0.43 | 3.76 | <0.001 |
| Radiologist, pathologist, and anesthesiologist (TE^‡^) to bed ratio |  |  |  |  |
| Radiologist, pathologist, and anesthesiologist (TE^‡^) to bed ratio | -0.1 | 0.75 | -0.14 | 0.892 |
| Radiology technician (FT^†^) to bed ratio |  |  |  |  |
| Radiology technician (FT^†^) to bed ratio | 0.62 | 0.45 | 1.38 | 0.166 |
| Registered nurse (FT^†^) to bed ratio |  |  |  |  |
| Registered nurse (FT^†^) to bed ratio | 0.08 | 0.09 | 0.89 | 0.375 |
| Respiratory therapist (FT^†^) to bed ratio |  |  |  |  |
| Respiratory therapist (FT^†^) to bed ratio | -1.26 | 1.91 | -0.66 | 0.509 |
| Total physician (TE^‡^) to bed ratio |  |  |  |  |
| Total physician (TE^‡^) to bed ratio | 0.46 | 0.16 | 2.91 | <0.001 |
| **Other** |  |  |  |  |
| Diversity orientation for clinical staff |  |  |  |  |
| Yes | 0.06 | 0.4 | 0.15 | 0.884 |
| Diversity strategy plan |  |  |  |  |
| Yes | 0.22 | 0.29 | 0.75 | 0.451 |
| Diversity training for all employees is required |  |  |  |  |
| Yes | 0.38 | 0.4 | 0.97 | 0.331 |
| Hospital has electronic health record |  |  |  |  |
| Yes, partially implemented | -0.22 | 0.14 | -1.58 | 0.115 |
| More foreign educated nurses hired to fill registered nurse vacancies |  |  |  |  |
| Less | 0.03 | 0.4 | 0.08 | 0.933 |
| More | -0.29 | 0.4 | -0.71 | 0.476 |
| Same | 0.28 | 0.24 | 1.17 | 0.243 |
| Number of new registered nurse graduates hired by bed |  |  |  |  |
| Number of new registered nurse graduates hired by bed | 0.55 | 1.4 | 0.39 | 0.696 |
| Strategic plan includes quality improvement goals for diverse patient populations |  |  |  |  |
| Yes | 0 | 0.4 | 0 | 1.000 |

*Data sources: New York’s Statewide Planning and Research Cooperative System (SPARCS) and American Hospital Association (AHA) Annual Survey Database

^†^Full-time

^‡^Total equivalent

^§^Full-time equivalent
